# Protective Mechanisms of *S. lycopersicum* Aqueous Fraction (Nucleosides and Flavonoids) on Platelet Activation and Thrombus Formation: * In Vitro*, *Ex Vivo* and *In Vivo* Studies

**DOI:** 10.1155/2013/609714

**Published:** 2013-09-16

**Authors:** Eduardo Fuentes, Jaime Pereira, Marcelo Alarcón, Claudio Valenzuela, Pablo Pérez, Luis Astudillo, Iván Palomo

**Affiliations:** ^1^Department of Clinical Biochemistry and Immunohematology, Faculty of Health Sciences, Interdisciplinary Excellence Research Program on Healthy Aging, Universidad de Talca, Talca, Chile; ^2^Centro de Estudios en Alimentos Procesados (CEAP), CONICYT-Regional, Gore Maule, R09I2001, Chile; ^3^Synthesis Laboratory, Chemical Institute of Natural Resources, Universidad de Talca, Chile; ^4^Department of Hematology-Oncology, School of Medicine, Pontificia Universidad Católica de Chile, Chile; ^5^Institute of Plant Biology and Biotechnology, Universidad de Talca, Chile

## Abstract

The purpose of this research was to investigate mechanisms of antiplatelet action of bioactive principle from *S. lycopersicum*. Aqueous fraction had a high content of nucleosides (adenosine, guanosine, and adenosine 5′-monophosphate) by HPLC analysis. Also aqueous fraction presented flavonoids content. Aqueous fraction inhibited platelet activation by 15 ± 6% (*P* < 0.05). Fully spread of human platelets on collagen in the presence of aqueous fraction was inhibited from 15 ± 1 to 9 ± 1 **μ**m^2^ (*P* < 0.001). After incubation of whole blood with aqueous fraction, the platelet coverage was inhibited by 55 ± 12% (*P* < 0.001). Platelet ATP secretion and aggregation were significantly inhibited by the aqueous fraction. At the same concentrations that aqueous fraction inhibits platelet aggregation, levels of sCD40L significantly decreased and the intraplatelet cAMP levels increased. In addition, SQ22536, an adenylate cyclase inhibitor, attenuated the effect of aqueous fraction toward ADP-induced platelet aggregation and intraplatelet level of cAMP. Platelet aggregation *ex vivo* (human study) and thrombosis formation *in vivo* (murine model) were inhibited by aqueous fraction. Finally, aqueous fraction may be used as a functional ingredient adding antiplatelet activities (nucleosides and flavonoids) to processed foods.

## 1. Introduction

Cardiovascular diseases (CVD) currently accounts for nearly half of noncommunicable diseases, accounting for 17.3 million deaths per year, a number that is expected to grow to >23.6 million by 2030 [1]. The platelet activation and subsequent platelet aggregation play an essential role in the development and progression of CVD [2, 3]. Thus, after platelets get activated and form aggregates, they increase the secretion of other potentially pro-atherogenic molecules, such as IL-1*β*, sCD40L, CCL5 and sP-selectin [4, 5]. Of this form, platelets are not only key mediators of thrombosis but also in inflammation by directly interactacting with leukocytes and others cell [6–8]. P-selectin and sCD40L platelet-derived seems to contribute to atherosclerotic lesion development and arterial thrombogenesis by forming large stable platelet-leukocyte aggregates [9, 10]. 

Interestingly, data from experimental, epidemiological and clinical studies indicates that fruit and vegetable (F&V) consumption have profound cardio-protective effects in the primary as well as secondary prevention of coronary heart disease, hence they are considered as cardiovascular friendly natural products [11]. Presently, in addition to their recognized high value in vitamins, minerals and dietary fiber, consuming F&V is associated with phytochemical content [12, 13], with specific actions on target functions [14]. In this sense, studies have demonstrated the platelet antiaggregant activity of fruits (red grapes, strawberries, kiwis and pineapples) and vegetables (garlic, onions, green onions, melons and tomatoes) [15, 16]. Among these elements of a healthy diet, consumption of tomato (*Solanum lycopersicum*) has been suggested to play an important role in preventing cardiovascular problems given to the phytochemicals with antioxidant activities [17–19].

However, very little is known on the mechanisms involved in the antiplatelet effects of *S. lycopersicum *and its bioactive compounds. The main aim of this work was to investigate mechanisms of antiplatelet and antithrombotic activities of aqueous fraction from *S. lycopersicum *and bioactive compounds that provide this activity.

## 2. Materials and Methods

### 2.1. Reagents

The agonists adenosine 5′-diphosphate bis (ADP), thrombin receptor-activating peptide 6 (TRAP-6), and arachidonic acid were from Sigma-Aldrich (St. Louis/MO, USA), while the collagen was obtained from Hormon-Chemie (Munich, Germany). Calcein-AM, bovine serum albumin (BSA), SQ22536 (adenylate cyclase inhibitor) adenosine, guanosine, adenosine 5′-monophosphate (AMP), gallic acid, quercetin, aluminium chloride (AlCl_3_), and Folin-Ciocalteu reagent were obtained Sigma-Aldrich (St. Louis/MO, USA), whereas luciferase/luciferin reagent was obtained from Chrono-Log Corp (Havertown, PA), and microfluidic chambers were from Bioflux (Fluxion, San Francisco, CA, USA). Annexin V FITC Apoptosis Kit was obtained from BD Pharmingen (BD Biosciences, San Diego, CA, USA).

### 2.2. Bioactive Extract and Fraction

Total extract and aqueous fraction from *Solanum lycopersicum* were obtained according to Fuentes et al. [20]. Briefly, the total extract was fractionated by liquid-liquid separation, obtaining an aqueous, ethyl acetate, and petroleum ether fractions. The aqueous fraction was lyophilized (Labconco, Freezone 6, Kansas City, MO, USA) and stored at −70°C until use. 

### 2.3. Total Phenolic and Total Flavonoid Content

Determination of total phenolic contents was determined using Folin–Ciocalteu reagent as adapted from Velioglu et al. [21], with slight modifications. In brief, 20 *µ*L of extract or fraction was mixed with 100 *µ*L of Folin-Ciocalteu reagent previously diluted with 1.58 mL of distilled water and allowed to stand at room temperature for 8 min; 300 *µ*L of sodium carbonate (20%) solution was added to the mixture. After 120 min at room temperature, absorbance was measured at 725 nm using a Unicam Helios Gamma spectrometer (Thermo Spectronic, Helios Gamma, Cambridge, UK) within the range of linearity (0.05–0.8 mM). Results were expressed as mg gallic acid equivalents in 100 g of the dried extract (mg GAE/100 g). The total flavonoid content of the extract or fraction was determined as follows [22]. Briefly, to 0.1 mL of sample, 2.35 mL methanol and 50 *µ*L of 5% AlCl_3_ ethanolic solution were added. After 1 h at room temperature, the absorbance was measured at 415 nm. Quercetin was used as a reference for the calibration curve, and results were expressed as mg quercetin equivalents per 100 g dried extract (mg QE/100 g). Total phenolic and total flavonoid contents were reported as mean ± SEM for at least three replications. 

### 2.4. HPLC Analysis

Analysis of the chemical profile of bioactive compound of aqueous fraction from *S. lycopersicum* was performed by HPLC Merck-Hitachi (La-Chrom, Tokyo, Japan) equipment consisting of an L-7100 pump, an L-7455 UV diode array detector, and a D-7000 chromatointegrator. HPLC-DAD analysis was carried out using a 250 mm × 4.60 mm i.d. and 5 *µ*m C18-RP Luna column (Phenomenex, Torrance, CA, USA) maintained at 25°C with a linear gradient solvent system consisting of 1% formic acid (A) and acetonitrile (B) as follows: 95–90% A over 10 min, 90–50% A over 10 min, 50–0% A over 5 min, and followed by 0–95% A from 25 to 30 min at a flow rate of 1 mL/min. UV spectra from 200 to 600 nm were recorded for peaks characterization. Standards of guanosine, AMP, and adenosine were used for HPLC-DAD identification and determination and dissolved in acetonitrile-formic acid (99 : 1, v/v) to prepare standard solutions ranging from 0.125 to 1 mg/mL. Aqueous fraction was lyophilized and then equilibrated to room temperature for 1 h and dissolved in acetonitrile-formic acid (99 : 1, v/v). All samples were filtered through a 5 *µ*m filter (Millipore Corporation, Bedford, MA), and then 20 *µ*L were injected. All samples were run in triplicate. 

### 2.5. Preparation of Human Platelet Suspensions

Venous blood samples were obtained from two healthy volunteers not taking aspirin or other nonsteroidal anti-inflammatory drugs for at least 10 day, in 3.2% citrate tubes (9 : 1 v/v) by phlebotomy with vacuum tube system (Becton Dickinson Vacutainer Systems, Franklin Lakes, NJ, USA). The protocol was approved by the Institutional Review Board Talca University in accordance with the Declaration of Helsinki. Informed consent was signed before the blood was drawn. The samples were gently mixed by 5-fold inversion and allowed to stand for 5 minutes. The tubes were centrifuged (DCS-16 Centrifugal Presvac RV) at 240 g for 10 minutes to obtain platelet-rich plasma (PRP). Platelet count in PRP was performed in a hematologic counter (Bayer Advia 60 Hematology System, Tarrytown, NY, USA). The original tubes were centrifuged at 650 g for 10 minutes to obtain the platelet-poor plasma (PPP). Finally, the PRP was adjusted to 200 × 10^9^ platelets/L with PPP. Washed platelets were prepared by adding 50 ng/mL PGE_1_ to the PRP, and platelets were then pelleted at 750 g for 10 minutes. Platelets were washed once in Tyrode-Hepes buffer containing 50 ng/mL PGE_1_ and 1 mmol/L EDTA, pH 7.4, and resuspended in Tyrode-Hepes to a concentration of 250 × 10^9^/L. Given the use of vacutainers for different experiments, samples were examined to discard the presence of platelet preactivation before isolation of PRP/washed platelets, by normal platelet count and absence of clots.

### 2.6. Flow Cytometry

Loss of platelet membrane phospholipid asymmetry with externalization of phosphatidylserine was assessed by the binding of annexin V by flow cytometry. To 480 *µ*L of citrated whole blood, collagen 1.5 *μ*g/mL and ADP 8 *μ*mol/L (final concentrations) were added for 10 min at 37°C, with stirring at 1.000 rpm. In each experiment, previous to the addition of platelet agonists, PRP was incubated with saline or aqueous fraction (1 mg/mL) for 10 min at room temperature. Briefly, 50 *μ*L of PRP was diluted with 150 *μ*L of binding buffer (10 mmol/L Hepes, 150 mmol/L NaCl, 5.0 mmol/L KCl, 1.0 mmol/L MgCl_2_, 2.0 mmol/L CaCl_2_, pH 7.4) and incubated for 25 min in the dark with 0.6 *μ*g/mL (final concentration) of annexin V-FITC (Sigma Chemical, St. Louis, MO) and anti-CD61-PE, (Pharmingen, San Diego, CA). The samples were acquired and analyzed in Accuri C6 flow cytometer (BD, Biosciences, USA). Platelet populations were gated on cell size using forward scatter (FSC) versus side scatter (SSC) and CD61 positivity to distinguish it from electronic noise. The light scatter and fluorescence channels were set at logarithmic gain, and 5000 events per sample were analyzed. Fluorescence intensities of differentially stained populations were expressed as mean channel value using the CSampler Software (BD Biosciences, USA).

### 2.7. Measurement of Platelet Aggregation and Secretion

Platelet aggregation was monitored by light transmission according to Born and Cross [23], using a lumi-aggregometer. Briefly, 480 *μ*L of PRP in the reaction vessel was preincubated with 20 *μ*L of saline, total extract (1 mg/mL), or aqueous fraction (1 mg/mL). After 3 min of incubation, 20 *μ*L of agonist was added to initiate platelet aggregation, which was measured for 6 min. ADP 8 *µ*mol/L, collagen 1.5 *μ*g/mL, TRAP-6 30 *µ*mol/L, and arachidonic acid 1 mmol/L were used as agonists. All measurements were performed in triplicate. The results of platelet aggregation (maximal amplitude [%], slope, area under the curve, and lag time [s]) were determined by the software AGGRO/LINK (Chrono-Log, Havertown, PA, USA). The inhibition of the maximal platelet aggregation by the fraction was expressed as a percentage with respect to control (saline).

Platelet secretion was determined by measuring the release of ATP using luciferin/luciferase reagent. Luciferin/luciferase (50 *μ*L) was added to 480 *μ*L of platelet suspension (PRP adjusted to 200 × 10^9^ platelets/L) within 2 min before stimulation. Platelet secretion was recorded in real time in a lumi-aggregometer (Chrono-Log, Havertown, PA, USA) at 37°C with stirring (1.000 rpm). To examine the effects on platelet secretion, platelets were preincubated with saline or aqueous fraction (1 mg/mL) for 3 min prior to the addition of ADP 8 *µ*mol/L [24].

### 2.8. Platelet Spreading Assay

Coverslips were coated with collagen (100 *μ*g/mL) and incubated at 37°C, for 60 minutes. Then they were rinsed with phosphate buffered saline (PBS), blocked with BSA 1% for 60 minutes at 37°C and finally washed with PBS to remove any unbound BSA. Washed platelets (5 × 10^9^ platelets/L) were labeled with calcein-AM (4 *µ*mol/L), preincubated with aqueous fraction (1 mg/mL) or apyrase (2 units/mL) for 1 hour at room temperature and then allowed to spread on collagen for 1 hour at 37°C [25]. After gently rinsing 3 times with PBS, spread platelets were mounted in Vectashield mounting medium (Vector Laboratories, Burlingame, CA). The images were acquired with Zeiss 40x oil-immersion lens (1.3 numeric aperture) and Photometrics SenSys camera (Photometrics, Tucson, AZ) from 4 consecutive fields and examined using a Carl Zeiss LSM 700 confocal microscope (Carl Zeiss, Oberkochen, Germany) and the argon-krypton laser at 488 nm to generate the fluorescent calcein signal detected between 490 nm and 530 nm. The images were analyzed using Image J software (version 1.26t, NIH, USA).

### 2.9. Platelet Adhesion and Aggregation under Controlled Flow

Experiments under flow were performed in a BioFlux 200 flow system (Fluxion, San Francisco, CA, USA) with high shear plates (48 wells, 0–20 dyne/cm^2^). Using manual mode in the BioFlux software, the microfluidic chambers were coated for 1 hour with 20 *μ*L of collagen 200 *µ*g/mL at a wall shear rate of 200 s^−1^. 

The plaque coating was allowed to dry at room temperature for one hour. The channels were perfused with PBS for 10 min at room temperature at wall shear rate of 200 s^−1^ to remove the interface. Then, the channels were blocked with BSA 5% for 10 min at room temperature at wall shear rate of 200 s^−1^. Whole blood anticoagulated with sodium citrate was labeled with calcein-AM (4 *μ*mol/L) and incubated at RT with saline or aqueous fraction (1 mg/mL). After one hour of incubation, the blood was added to the inlet of the well, and chambers were perfused for 10 min at room temperature at a wall shear rate of 1000 s^−1^. The plates were mounted on the stage of an inverted fluorescence microscope (TE200, NIKON, Japan) [26].

Platelet deposition was observed and recorded in real time (30 frames per min) with a CCD camera (QICAM, QIMaging, Surrey, BC, Canada). We used bright field and fluorescence microscopy for real-time visualization of platelet adhesion and aggregation in flowing blood. For each flow experiment, fluorescence images were analyzed off-stage by quantifying the area covered by platelets with the Image J software (version 1.26t, NIH, USA). In each field, the area covered by platelets was quantified.

### 2.10. Measurement of cAMP Levels in Platelets

The effect of aqueous fraction on platelet levels of cAMP was evaluated in PRP samples (500 *µ*L) following 5 min incubation without stirring. Platelet reactions were stopped with 150 *µ*L of ice-cold 10% trichloroacetic acid. Precipitated proteins were removed by centrifugation at 2.000 g for 15 min at 4°C. Following addition of 150 *µ*L of HCl 1 mol/L, the supernatant was submitted to 6 ether extractions v/v and lyophilized. Samples were stored at −70°C until assay. Before determination, the powder was dissolved in 200 *µ*L of PBS, pH 6.2. cAMP Direct Immunoassay Kit (BioVision Research Products, Mountain View, CA, USA) was employed.

### 2.11. Effect of SQ22536 on ADP-Induced Platelet Aggregation and Intraplatelet Level of cAMP

To elucidate whether antiplatelet effect of aqueous fraction was mediated by stimulation of cAMP production, PRP was pretreated with SQ22536 (250 and 500 *µ*mol/L) for 3 min and followed by the addition of aqueous fraction (1 mg/mL). Platelet aggregation was measured by addition of ADP 8 *µ*mol/L. Platelets pretreated with SQ22536 (250 and 500 *µ*mol/L) and followed by the addition of aqueous fraction (1 mg/mL) were prepared for measurement of intraplatelet level of cAMP as described above.

### 2.12. Measurement of sCD40L Levels

Soluble CD40 ligand (sCD40L) was determined using a Human sCD40-Ligand Quantikine kit (R&D systems, Minneapolis, MN). Briefly, washed platelet was pretreated with saline, aqueous fraction (1 mg/mL), or aspirin 0.3 mmol/L for 15 min at 37°C and then stimulated by thrombin (2 U/mL) for 45 at 37°C. Finally, the supernatants were collected following centrifugation at 11.000 g for 10 min at 4°C and stored at −70°C prior to sCD40L measurements by ELISA as described earlier [27].

### 2.13. Thrombus Formation in Murine Model

This model is an adaptation of one previously described [28]. Briefly, mice (12–16 weeks old) were anesthetized with a combination of tribromoethanol (270 mg/kg) and xylazine (13 mg/kg). The mesentery was exposed by central incision in the abdomen, permitting visualization of thrombus development in mesenteric artery. The mice were injected with rose bengal (Sigma, St Louis, MO) through tail vein injection in a volume of 0.1 mL at a concentration of 50 mg/kg. Just after injection, a 1.5-mW green light laser (532 nm) was applied to the desired site of mesenteric artery, and blood flow was monitored for 60 minutes. Stable occlusion was defined as a blood flow of 0 mL/min for 3 minutes. Control group (saline, *n* = 5), acetylsalicylic acid (200 mg/Kg, *n* = 5), or aqueous fraction (200 mg/kg, *n* = 5) was administered intraperitoneally 30 min before experiment.

### 2.14. Measurement of Platelet Aggregation *Ex Vivo*

Six apparently healthy volunteers (3 men and 3 women; age 23 to 30 years), with no history of haemostatic disorders, were instructed to abstain from consuming drugs known to affect platelet function for a 10-day period before their participation in the study. Written informed consent was obtained from all subjects. The study was approved by the Institutional Review Board Talca University. Platelet aggregation induced by ADP (4 *µ*mol/L) was studied before (baseline) and four hours after oral administration of tomato aqueous fraction (70 mg/kg) diluted in 50 mL of orange juice. 

### 2.15. Statistical Analysis

All data are expressed as mean ± standard error of mean (SEM). Differences between the different groups were analysed by Student's paired or unpaired *t*-test and one-way analysis of variance with Duncan's post-hoc test using SPSS version 17.0. The statistical significance level was set up at *P* < 0.05.

## 3. Results

### 3.1. Total Phenolic and Total Flavonoid Contents

The total phenols presented statistically significant differences and were in the following order: aqueous extract (11 ± 1 mg GAE/100 g) > aqueous fraction (6.8 ± 0.9 mg GAE/100 g) (*P* < 0.05), and the total flavonoids presented the similar order, but no significant differences: aqueous extract (1.74 ± 0.3 mg QE/100 g) > aqueous fraction (1.52 ± 0.5 mg QE/100 g) (*P* > 0.05).

### 3.2. Chromatographic Analysis of *S. lycopersicum* Aqueous Fraction

**Figure 1:**
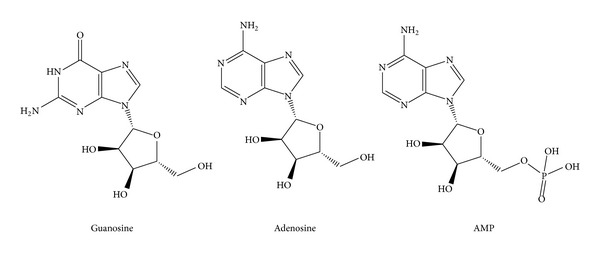
Bioactive compounds indentified in aqueous fraction from *S. lycopersicum* by HPLC.

HPLC analysis of aqueous fraction from *S. lycopersicum* revealed a group of nucleosides, which have been known as adenosine, guanosine, and AMP (Figure 1). Based on HPLC determination, the content of nucleosides in aqueous fraction was in the following increasing order: guanosine (5.4 mg/g dried), AMP (9.9 mg/g dried), and adenosine (155 mg/g dried). Similar compounds have been reported by ^1^H-NMR using total tomato extract of the same plant [29].

### 3.3. Total Tomato Extract Inhibits Platelet Aggregation Induced by Different Agonists

**Figure 2:**
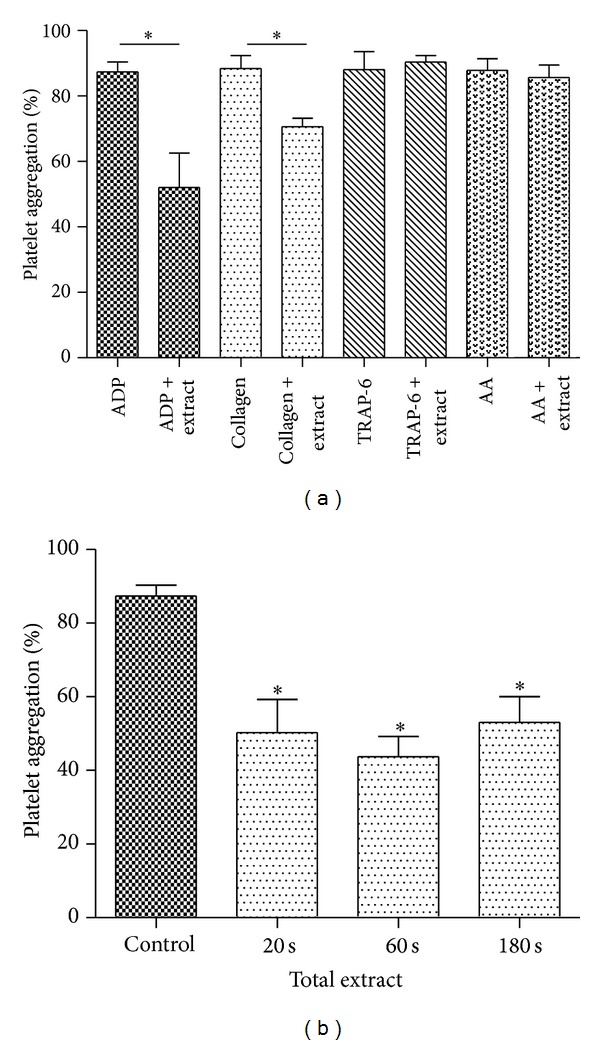
Effects of total extract on platelet aggregation. (a) PRP was incubated with saline or total extract (1 mg/mL) as indicated for 3 min, prior to measuring human platelet aggregation induced by ADP 8 *µ*M, collagen 1.5 *μ*g/mL, AA (arachidonic acid 1 mM) or TRAP-6 30 *µ*M, and (b) PRP was incubated with saline or total extract (1 mg/mL) for 20, 60, and 180 seconds, prior to measuring human platelet aggregation induced by ADP 8 *µ*M. The graph depicts the mean ± SEM of *n* = 3 experiments. *P < 0.05. PRP: platelet-rich plasma.

To first explore the potential antiplatelet activity of *S. lycopersicum,* a total extract was tested on platelet aggregation induced by different agonists. The effect of total extract from fully mature tomatoes on platelet aggregation induced by ADP, collagen, TRAP-6, and arachidonic acid is shown in Figure 2. The total extract (1 mg/mL) inhibited ADP- and collagen-induced platelet aggregation by 36 ± 10% and 19 ± 4%, respectively (*P* < 0.05 versus control) (Figure 2(a)). The time dependency of this effect was tested by preincubation of PRP with the extract at different times (20, 60, and 180 seconds) before the addition of ADP 8 *µ*mol/L. As observed in Figure 2(b) the inhibitory activity was significant even after 20 seconds of incubation with an average of 34 ± 8% as compared to that the control. 

### 3.4. Aqueous Fraction of *S. lycopersicum* Inhibits Several Platelet Activation Events

**Figure 3:**
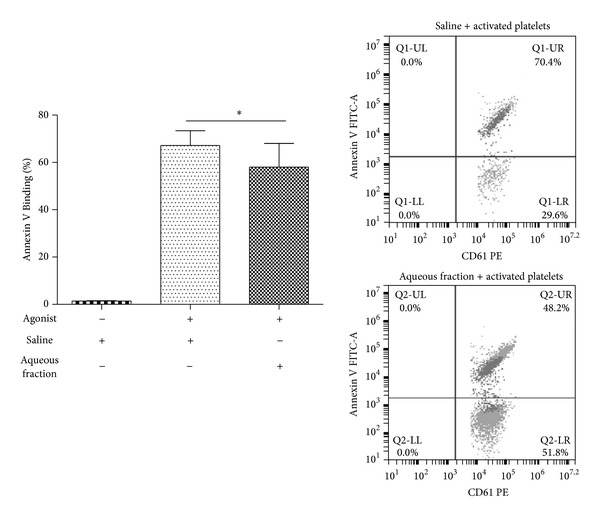
Aqueous fraction inhibited platelet activation. Platelet activation was measured by percentage of annexin V binding (flow cytometry). PRP was incubated with saline or aqueous fraction (1 mg/mL) for 10 min, prior to measuring human platelet activation induced by agonist (collagen/ADP). The graph depicts the mean ± SEM of *n* = 3 experiments. *P < 0.05. PRP: platelet-rich plasma.

**Figure 4:**
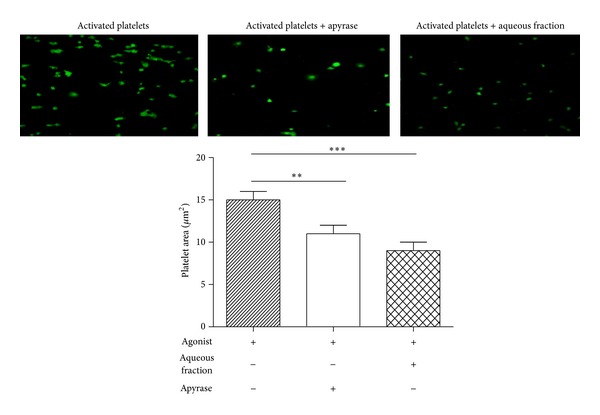
Effect of aqueous fraction on spreading of human platelets on collagen-coated surfaces. In this experiment, washed platelets were employed. Platelet area (*µ*m^2^) mean ± SEM was acquired from 4 consecutive fields using a Carl Zeiss LSM 700 confocal microscope. **P < 0.01 and ***P < 0.001.

**Figure 5:**
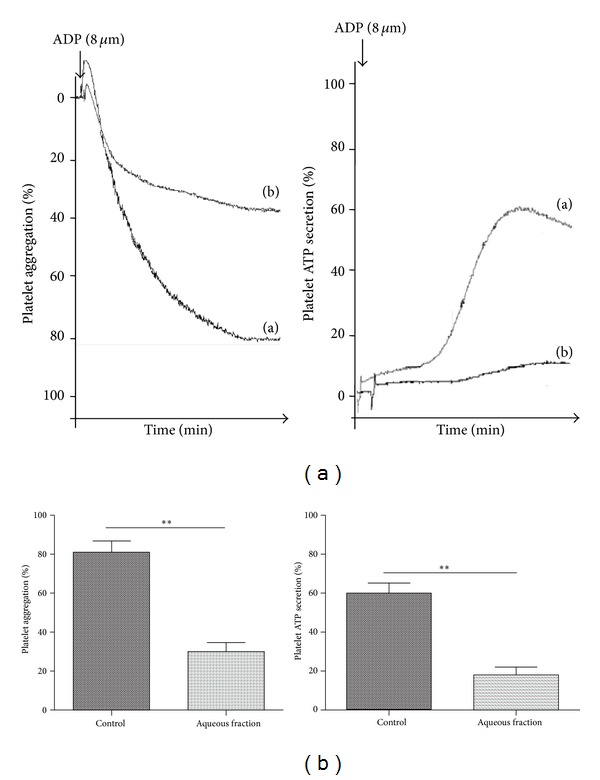
Aqueous fraction inhibited platelet aggregation and ATP secretion. (a) saline and (b) aqueous fraction (1 mg/mL). Luciferin/luciferase reagent and then ADP 8 *µ*M were added to PRP to induce aggregation and secretion, which were recorded in real time using the lumi-aggregometer. Each aggregation curve is representative of multiple traces obtained from three separate platelet donors. The graph depicts the mean ± SEM of *n* = 3 experiments. **P < 0.01. PRP: platelet-rich plasma.

We investigated the antiplatelet effects of the aqueous fraction of *S. lycopersicum* obtained by liquid-liquid separation by testing its activity on different activation-dependent events in human platelets. Activated platelets expose phosphatidylserine (PS), which is a key phenomenon for generating a burst of thrombin essential to thrombus growth. The aqueous fraction inhibited collagen/ADP-induced externalization of PS assessed by annexin V binding by 15 ± 6% (*P* < 0.05) (Figure 3). It is well established that platelets undergo a dramatic change in morphology upon binding to immobilized adhesive proteins [30]. To expand the understanding of the effects of aqueous fraction as an inhibitor of collagen-mediated inside-out signaling, we assessed its effect on platelet spreading on collagen-coated surfaces. The fully spreading of human platelets on immobilized collagen in the presence of aqueous fraction was inhibited from 15 ± 1 to 9 ± 1 *µ*m^2^ (*P* < 0.001) (Figure 4). ADP-induced platelet aggregation and ATP release were inhibited by 54 ± 13% and 52 ± 5%, respectively (*P* < 0.05) (Figure 5). 

### 3.5. Aqueous Fraction of *S. lycopersicum* Impairs Platelet Adhesion on Immobilized Collagen under Flow Conditions

**Figure 6:**
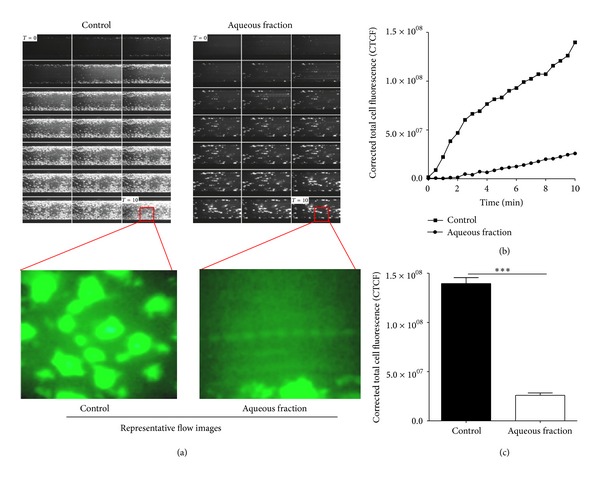
Effect of aqueous fraction on collagen-induced platelet adhesion and aggregation under arterial flow conditions. Citrate-anticoagulated blood was preincubated with saline or aqueous fraction (1 mg/mL) for 1 hour and then was perfused over plaque-coated surfaces for 10 min at room temperature at a shear rate of 1000 s^−1^. (a) Time lapse of 10 min at 1000 s^−1^, at 30 sec intervals, representative flow images at 40x, (b) the intensity (CTCF) over a time lapse, and (c) bar diagram (values are mean ± SEM; *n* = 3). ***P < 0.001.

The effects of aqueous fraction on platelet adhesion/aggregation to immobilized collagen under arterial flow conditions are shown in Figure 6. After perfusion of citrate-anticoagulated blood over collagen-coated plaque surfaces at 37°C with a wall shear rate of 1000 s^−1^ for 10 min, rapid platelet adhesion and aggregate formation were observed (Figure 6 and supplemental video 1). Aqueous fraction reduced collagen-induced platelet adhesion and aggregate formation under controlled flow. After aqueous fraction incubation of blood, the platelet coverage was inhibited by 55 ± 12% (*P* < 0.001) (Figure 6 and supplemental video 2).

### 3.6. Effect of Aqueous Fraction of *S. lycopersicum* on Intraplatelet Levels of cAMP

Because we have recently demonstrated that the aqueous fraction from *S. lycopersicum* contains significant amounts of adenosine [20], we hypothesized that the antiplatelet effect could be exerted by rising cAMP levels. At 1 mg/mL the aqueous fraction significantly increased the intraplatelet levels of cAMP from 3.5 ± 1 to 20 ± 2 pmol/10^8^ platelets (*P* < 0.05). 

### 3.7. Effect of SQ22536 on ADP-Induced Platelet Aggregation and Intraplatelet Level of cAMP

cAMP production through adenylate cyclase activation has been shown to inhibit platelet aggregation [31]. Interestingly we found that SQ22536 attenuated the increase of intraplatelet levels of cAMP by aqueous fraction from 20 ± 2 to 5 ± 2 and 3 ± 1 pmol/10^8^ platelets at concentrations of 250 and 500 *µ*mol/L, respectively (*P* < 0.05). 

**Figure 7:**
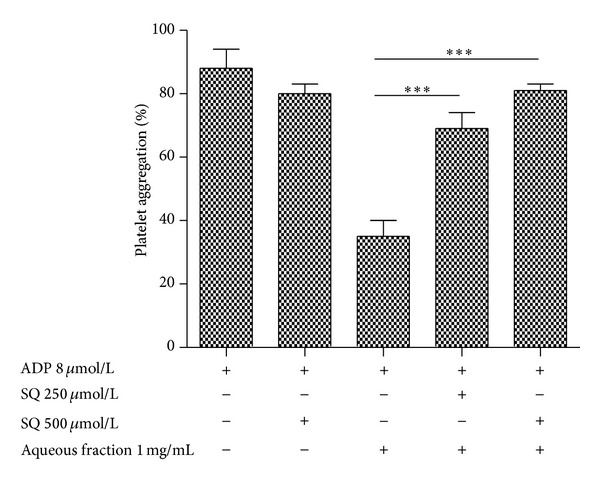
Effect of SQ22536 on platelet aggregation induced by ADP. PRP suspension was incubated with ADP or aqueous fraction plus ADP or pretreated with SQ22536 for 3 min, followed by addition of aqueous fraction and ADP. The graph depicts the mean ± SEM of *n* = 3 experiments. ***P < 0.001. PRP: platelet-rich plasma.

We therefore tested whether SQ22536 could reverse the inhibitory effect of aqueous fraction (1 mg/mL) toward ADP-induced platelet aggregation. Of this forma, SQ22536 was able to reverse the inhibition of aqueous fraction (1 mg/mL) on ADP-induced platelet aggregation. Thus, SQ22536 attenuated the inhibitory effect of aqueous fraction toward ADP-induced platelet aggregation by 34 and 52% at concentrations of 250 and 500 *µ*mol/L, respectively (*P* < 0.001) (Figure 7). As a control, SQ22536 by itself did not show effect on ADP- (8 *µ*mol/L) induced platelet (Figure 7). 

### 3.8. Effect on Levels of sCD40L

**Figure 8:**
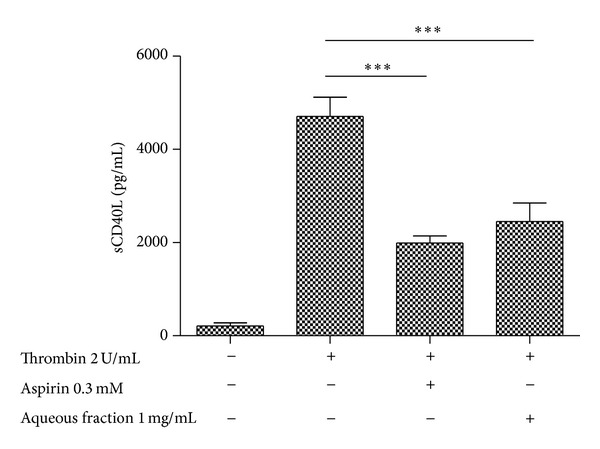
Effect of aqueous fraction on release of sCD40L from platelets. In this experiment, washed platelets were employed. The graph depicts the mean ± SEM of *n* = 3 experiments. ***P < 0.001.

As platelets are considered the major source of sCD40L in the blood [32], we examined the effect of aqueous fraction on release of sCD40L. As observed in Figure 8, we found that aqueous fraction significantly reduced thrombin-induced sCD40L release from platelets (*P* < 0.001). Even aqueous fraction had a similar effect that aspirin.

### 3.9. Effect on Arterial Thrombus Formation *In Vivo*

**Figure 9:**
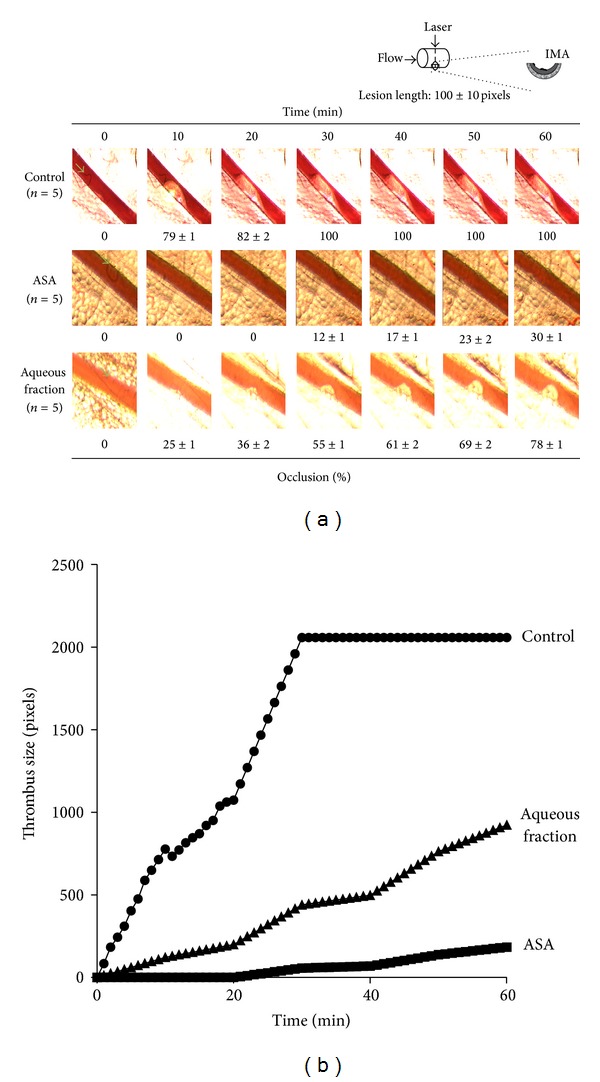
Aqueous fraction inhibited arterial thrombosis formation. (a) Representative images of thrombus formation after laser-injured vascular injury in mouse mesenteric artery, saline control group (*n* = 5), ASA (acetylsalicylic acid) (200 mg/Kg, *n* = 5), and aqueous fraction (200 mg/Kg, *n* = 5) to 60 min and (b) representative time course changes of thrombus growth rate.

All animals in this study showed similar physiological values for rectal temperature before and after thrombosis model among groups (data not shown). To study arterial thrombus development in mesenteric artery, anesthetized animals were administered with saline, acetylsalicylic acid, or aqueous fraction at a dose of 200 mg/kg body weight by intraperitoneal injection. We examined the effect of aqueous fraction on arterial thrombus formation, as shown in Figure 9. The arterial thrombosis formation to 60 min was inhibited from 98 ± 2 to 30 ± 1% (*n* = 5, *P* < 0.001) occlusion by pretreatment with antiplatelet agents such as acetylsalicylic acid. During the time in which blood flow was monitored, aqueous fraction significantly inhibited arterial occlusion. Administration of aqueous fraction (occlusion: 78 ± 1%, *n* = 5) showed significant reductions in occlusion size compared with the negative control to 60 min (occlusion: 98 ± 2%, *n* = 5) (*P* < 0.05). Over time, both acetylsalicylic acid and aqueous fraction present a similar kinetics of inhibition of arterial thrombosis formation (Figure 9(b)). 

### 3.10. Acute Administration of Aqueous Tomato Fraction Inhibits ADP-Induced Platelet Aggregation in Healthy Subjects

**Figure 10:**
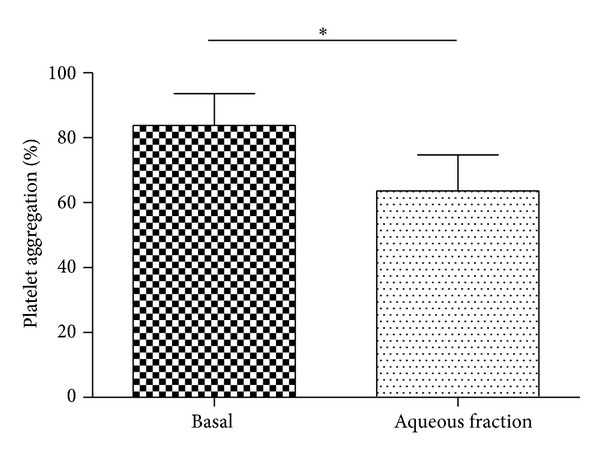
Administration of aqueous fraction inhibiting ADP-induced platelet aggregation in healthy subjects. Platelet aggregation in PRP induced by ADP 4 *µ*M was studied before (basal) and four hours after oral administration of aqueous fraction (70 mg/kg). The graph depicts the mean ± SEM of *n* = 6 (each in triplicate) healthy volunteers. *P < 0.05. PRP: platelet-rich plasma.

Platelet aggregation induced by ADP was significantly reduced as compared with baseline values after intake of aqueous tomato fraction by healthy human subjects. The average baseline platelet aggregation for the whole group was 86 ± 7%. In 6 healthy subjects who received the aqueous fraction, four hours after treatment we observed a significant inhibition of platelet aggregation induced by ADP for 19 ± 7% compared with respect to basal (*P* < 0.05) (Figure 10). The volunteers after treatment did not reporte side effects, such as dizziness, stomach pain, and fever.

## 4. Discussion

In this study, we have demonstrated that aqueous fraction of *S. lycopersicum* displayed a range of antiplatelet activities targeting different platelet activation responses *in vitro*, *ex vivo* and *in vivo*. This activity is mainly due to the presence of bioactive compounds (nucleosides and flavonoids) identified in aqueous fraction with antiplatelet activity [33–35]. 

Given the central role played by platelets in the pathogenesis of atherothrombosis, a variety of antiplatelet agents have been developed to prevent thrombotic ischemic events [36]. Although antiplatelet drugs have being used widely in the management of acute episodes and secondary prevention, its effectiveness in primary prevention is still a matter of debate, and CVDs still represent the leading causes of morbidity and mortality, worldwide [37]. In this sense, a dietary strategy for the prevention of cardiovascular diseases appears to be demanding and highly relevant in preventive medicine [37]. In fact, epidemiological studies have provided evidence of a protective role of healthy diets in the prevention of cardiovascular diseases [38]. The protective effects of F&V may be originated from several phytochemicals present in foods. We have recently demonstrated that *S. lycopersicum *exerts antiplatelet activity through the inhibition of platelet aggregation induced by ADP and collagen [39, 40]. In the same line, we found that total extract of *S. lycopersicum* was thermally stable in the temperature range of 20 to 100°C, and both acid and alkali did not affect inhibition of platelet aggregation induced by ADP [41]. In the present study, we extended these observations exploring the antiplatelet effects of an aqueous fraction of *S. lycopersicum*.

The mechanisms involved in the inhibition of platelet aggregation by aqueous fraction from *S. lycopersicum* have not been yet elucidated. In the present study, the aqueous fraction inhibited platelet activation induced by collagen and ADP. When platelets adhere to collagen, a ligand-binding-induced signal is generated, leading to platelet spreading that render adherent platelets resistant to the shear forces at the site of vascular damage. To study whether *S. lycopersicum* fraction was able to inhibit spreading, platelets were allowed to adhere to collagen, and spreading was evaluated by differential interference contrast and confocal microscopy. As shown in Figure 4 aqueous fraction prevented platelet spreading onto collagen under static conditions. 

Under conditions of arterial shear, platelets tethered to immobilized collagen. Aqueous fraction significantly reduced platelet adhesion and aggregation to collagen as compared with that in the negative control. In addition, we found that the same doses of aqueous fraction that caused a significant inhibition of platelet secretion and aggregation also produced a substantial increase in platelet cAMP levels. In this study SQ22536, an adenylate cyclase inhibitor, at different concentrations was able to attenuate the platelet antiaggregant activity and increase of cAMP by aqueous fraction. The inhibition of platelet aggregation by aqueous fraction is thought to be mediated by the stimulation of adenylate cyclase with increase of intraplatelet cAMP concentrations. Thus, this study provides the first documentation that aqueous fractions from *S. lycopersicum* have the ability to markedly raise human platelet cAMP levels, which contradicts previous results of Lazarus and Garg [35]. The anterior can be explained by the presence of nucleosides and flavonoids in aqueous fraction. Nucleosides and flavonoids act on adenylate cyclase participation with increase of cAMP levels (antiplatelet activity) [42, 43]. Also O'Kennedy established that tomato extracts inhibit platelet P-selectin expression after being stimulated by ADP [34]. Thus the relationship between cAMP levels and P-selectin expression is because cAMP via activation of PKA is capable of inhibiting platelet P-selectin expression [44, 45]. However, also it is possible that flavonoids present in the aqueous fraction can inhibit platelet function by another mechanism of action.

Moreover, studies establish that the inhibitory effect of adenosine or fraction from tomato rich in adenosine on platelet aggregation disappears after the addition of adenosine-deaminase [33, 46]. However, inosine (naturally occurring nucleoside degraded from adenosine) possesses antiplatelet activity *in vitro* and *in vivo* [47].

Reports in the last decade have described that the secretion of proinflammatory molecules (sCD40L, RANTES, sP-selectin, etc.) by platelet exacerbates the inflammatory response and generates a transition to an unstable plaque [7]. Thus, high levels of sCD40L have been associated with platelet activation, suggesting a prognostic value in patients with advanced atherosclerosis [48]. The present study indicates that aqueous fraction not only inhibits platelet function, but also decreases the inflammatory component of activated platelets, through a lower release of sCD40L.

Inhibition of platelet aggregation by drugs may represent an increased therapeutic possibility for such diseases. Platelet aggregation plays a pathophysiological role in arterial thrombosis [49]. Because the antiplatelet activity of aqueous fraction, it was studied on thrombus formation using a murine arterial thrombus formation *in vivo*, which causes formation of a mixed platelet/fibrin thrombus [50]. In the arterial thrombosis model, aqueous fraction inhibited arterial thrombus growth presenting similar kinetics of inhibition that aspirin, a classical reference drug.

The consumption of aqueous fraction components led to a reduction of 20% from baseline platelet aggregation response in the human study. The solution administered contained 4.9 g of aqueous fraction (e.g., for 70 Kg of bodyweight), which is equivalent to the quantity of aqueous fraction found in 6 fresh tomatoes. This amount of fresh tomatoes, which contains the active fraction required to reduce platelet function, is similar to that reported by O'Kennedy et al. [34]. 

In the present study, besides the known platelet antiaggregant activity, we demonstrated that the aqueous fraction from *S. lycopersicum* strongly inhibited platelet activation events. The broad range of antiplatelet effects found in the aqueous fractions of *S. lycopersicum* may render this functionally active principle in potents inhibitors of platelet function (nucleosides and flavonoids) with a potential preventive effect on thrombus formation.

## 5. Conclusion

The mechanisms underlying the antiplatelet action of aqueous fraction from *S. lycopersicum* seem to be related to the inhibition of platelet function through a substantial increase of intraplatelet levels of cAMP with significative effect on atheroinflammation/atherothrombosis. These effects, in terms of primary prevention, could modify cardiovascular risk without any of the side effects normally associated with antiplatelet drugs. Moreover, *S. lycopersicum* may constitute a functional ingredient adding antiplatelet activities to processed foods. 

## References

[1] Laslett LJ, Alagona P, Clark BA (2012). The worldwide environment of cardiovascular disease: prevalence, diagnosis, therapy, and policy issues: a report from the American College of Cardiology. *Journal of the American College of Cardiology*.

[2] Palomo I, Toro C, Alarcón M (2008). The role of platelets in the pathophysiology of atherosclerosis (Review). *Molecular Medicine Reports*.

[3] Gregg D, Goldschmidt-Clermont PJ (2003). Cardiology patient page. Platelets and cardiovascular disease. *Circulation*.

[4] Blair P, Flaumenhaft R (2009). Platelet *α*-granules: basic biology and clinical correlates. *Blood Reviews*.

[5] Cognasse F, Boussoulade F, Chavarin P (2006). Release of potential immunomodulatory factors during platelet storage. *Transfusion*.

[6] da Costa Martins PA, van Gils JM, Mol A, Hordijk PL, Zwaginga JJ (2006). Platelet binding to monocytes increases the adhesive properties of monocytes by up-regulating the expression and functionality of *β*1 and *β*2 integrins. *Journal of Leukocyte Biology*.

[7] da Costa Martins P, van den Berk N, Ulfman LH, Koenderman L, Hordijk PL, Zwaginga JJ (2004). Platelet-monocyte complexes support monocyte adhesion to endothelium by enhancing secondary tethering and cluster formation. *Arteriosclerosis, Thrombosis, and Vascular Biology*.

[8] Fuentes QE, Fuentes QF, Andres V (2013). Role of platelets as mediators that link inflammation and thrombosis in atherosclerosis. *Platelets*.

[9] Burger PC, Wagner DD (2003). Platelet P-selectin facilitates atherosclerotic lesion development. *Blood*.

[10] Chandler AB, Earhart AD, Speich HE (2010). Regulation of CD40L (CD154) and CD62P (p-selectin) surface expression upon GPIIb-IIIa blockade of platelets from stable coronary artery disease patients. *Thrombosis Research*.

[11] Shukla SK, Gupta S, Ojha SK, Sharma SB (2010). Cardiovascular friendly natural products: a promising approach in the management of CVD. *Natural Product Research*.

[12] Carlsen MH, Halvorsen BL, Holte K (2010). The total antioxidant content of more than 3100 foods, beverages, spices, herbs and supplements used worldwide. *Nutrition Journal*.

[13] Lako J, Trenerry VC, Wahlqvist M, Wattanapenpaiboon N, Sotheeswaran S, Premier R (2007). Phytochemical flavonols, carotenoids and the antioxidant properties of a wide selection of Fijian fruit, vegetables and other readily available foods. *Food Chemistry*.

[14] Badimon L, Vilahur G, Padro T (2010). Nutraceuticals and atherosclerosis: human trials. *Cardiovascular Therapeutics*.

[15] Pierre S, Crosbie L, Duttaroy AK (2005). Inhibitory effect of aqueous extracts of some herbs on human platelet aggregation in vitro. *Platelets*.

[16] Torres-Urrutia C, Guzmán L, Schmeda-Hirschmann G (2011). Antiplatelet, anticoagulant, and fibrinolytic activity in vitro of extracts from selected fruits and vegetables. *Blood Coagulation and Fibrinolysis*.

[17] Palomo I, Gutiérrez M, Astudillo L (2009). Efecto antioxidante de frutas y hortalizas de la zona central de Chile. *Revista Chilena De Nutrición*.

[18] Rao AV, Rao LG (2007). Carotenoids and human health. *Pharmacological Research*.

[19] Fuentes E, Alarco'n M, Astudillo L, Valenzuela C, Gutiérrez M, Palomo I (2013). Protective mechanisms of guanosine from *Solanum lycopersicum* on agonist-induced platelet activation: role of sCD40L. *Molecules*.

[20] Fuentes E, Castro R, Astudillo L (2012). Bioassay-guided isolation and HPLC determination of bioactive compound that relate to the anti-platelet activity (adhesion, secretion and aggregation) from *Solanum lycopersicum*. *Evidence-Based Complementary and Alternative Medicine*.

[21] Velioglu YS, Mazza G, Gao L, Oomah BD (1998). Antioxidant activity and total phenolics in selected fruits, vegetables, and grain products. *Journal of Agricultural and Food Chemistry*.

[22] Cuello S, Alberto MR, Zampini IC, Ordoñez RM, Isla MI (2011). Comparative study of antioxidant and anti-inflammatory activities and genotoxicity of alcoholic and aqueous extracts of four Fabiana species that grow in mountainous area of Argentina. *Journal of Ethnopharmacology*.

[23] Born GV, Cross MJ (1963). The aggregation of blood platelets. *The Journal of Physiology*.

[24] Li Z, Zhang G, Le Breton GC, Gao X, Malik AB, Du X (2003). Two waves of platelet secretion induced by thromboxane A2 receptor and a critical role for phosphoinositide 3-kinases. *The Journal of Biological Chemistry*.

[25] Boylan B, Gao C, Rathore V, Gill JC, Newman DK, Newman PJ (2008). Identification of FcgammaRlla as the ITAM-bearing receptor mediating alphallbbeta3 outside-in integrin signaling in human platelets. *Blood*.

[26] Conant CG, Schwartz MA, Nevill T, Ionescu-Zanetti C (2009). Platelet adhesion and aggregation under flow using microfluidic flow cells. *Journal of Visualized Experiments*.

[27] Antczak AJ, Singh N, Gay SR, Worth RG (2010). IgG-complex stimulated platelets: a source of sCD40L and RANTES in initiation of inflammatory cascade. *Cellular Immunology*.

[28] Przyklenk K, Whittaker P (2007). Adaptation of a photochemical method to initiate recurrent platelet-mediated thrombosis in small animals. *Lasers in Medical Science*.

[29] Le Gall G, Colquhoun IJ, Davis AL, Collins GJ, Verhoeyen ME (2003). Metabolite profiling of tomato (*Lycopersicon esculentum*) using ^1^H NMR spectroscopy as a tool to detect potential unintended effects following a genetic modification. *Journal of Agricultural and Food Chemistry*.

[30] Inoue O, Suzuki-Inoue K, Dean WL, Frampton J, Watson SP (2003). Integrin *α*2*β*1 mediates outside-in regulation of platelet spreading on collagen through activation of Src kinases and PLC*γ*2. *Journal of Cell Biology*.

[31] Feijge MAH, Ansink K, Vanschoonbeek K, Heemskerk JWM (2004). Control of platelet activation by cyclic AMP turnover and cyclic nucleotide phosphodiesterase type-3. *Biochemical Pharmacology*.

[32] Danese S, A Katz JA, Saibeni S (2003). Activated platelets are the source of elevated levels of soluble CD40 ligand in the circulation of inflammatory bowel disease patients. *Gut*.

[33] Dutta-Roy AK, Crosbie L, Gordon MJ (2001). Effects of tomato extract on human platelet aggregation in vitro. *Platelets*.

[34] O’Kennedy N, Crosbie L, van Lieshout M, Broom JI, Webb DJ, Duttaroy AK (2006). Effects of antiplatelet components of tomato extract on platelet function in vitro and ex vivo: a time-course cannulation study in healthy humans. *American Journal of Clinical Nutrition*.

[35] Lazarus SA, Garg ML (2004). Tomato extract inhibits human platelet aggregation in vitro without increasing basal cAMP levels. *International Journal of Food Sciences and Nutrition*.

[36] Collins B, Hollidge C (2003). Antithrombotic drug market. Market indicators. *Nature Reviews Drug Discovery*.

[37] Raju NC, Eikelboom JW (2012). The aspirin controversy in primary prevention. *Current Opinion in Cardiology*.

[38] Kris-Etherton PM, Hecker KD, Bonanome A (2002). Bioactive compounds in foods: their role in the prevention of cardiovascular disease and cancer. *American Journal of Medicine*.

[39] Palomo I, Fuentes E, Padró T, Badimon L (2012). Platelets and atherogenesis: platelet anti-aggregation activity and endothelial protection from tomatoes (*Solanum lycopersicum* L.). *Experimental and Therapeutic Medicine*.

[40] Fuentes EJ, Astudillo LA, Gutiérrez MI (2012). Fractions of aqueous and methanolic extracts from tomato (*Solanum lycopersicum* L.) present platelet antiaggregant activity. *Blood Coagulation and Fibrinolysis*.

[41] Lebon G, Warne T, Edwards PC (2011). Agonist-bound adenosine A2A receptor structures reveal common features of GPCR activation. *Nature*.

[42] Bojić M, Debeljak Ž, Tomčiić M, Medić-Šari M, Tomić S (2011). Evaluation of antiaggregatory activity of flavonoid aglycone series. *Nutrition Journal*.

[43] Libersan D, Rousseau G, Merhi Y (2003). Differential regulation of P-selectin expression by protein kinase A and protein kinase G in thrombin-stimulated human platelets. *Thrombosis and Haemostasis*.

[44] Minamino T, Kitakaze M, Asanuma H (1998). Endogenous adenosine inhibits P-selectin-dependent formation of coronary thromboemboli during hypoperfusion in dogs. *Journal of Clinical Investigation*.

[45] Altman R, Rouvier J, Weisenberger H (1985). Identification of platelet inhibitor present in the melon (Cucurbitacea cucumis melo). *Thrombosis and Haemostasis*.

[46] Hsiao G, Lin KH, Chang Y (2005). Protective mechanisms of inosine in platelet activation and cerebral ischemic damage. *Arteriosclerosis, Thrombosis, and Vascular Biology*.

[47] Wagner DD, Burger PC (2003). Platelets in Inflammation and Thrombosis. *Arteriosclerosis, Thrombosis, and Vascular Biology*.

[48] Setianto BY, Hartopo AB, Gharini PPR, Anggrahini DW, Irawan B (2010). Circulating soluble CD40 ligand mediates the interaction between neutrophils and platelets in acute coronary syndrome. *Heart and Vessels*.

[49] Barrett NE, Holbrook L, Jones S (2008). Future innovations in anti-platelet therapies. *British Journal of Pharmacology*.

[50] Hechler B, Nonne C, Eckly A (2010). Arterial thrombosis: relevance of a model with two levels of severity assessed by histologic, ultrastructural and functional characterization. *Journal of Thrombosis and Haemostasis*.

